# Characterization and implementation of the MarathonRT template-switching reaction to expand the capabilities of RNA-seq

**DOI:** 10.1261/rna.080032.124

**Published:** 2024-11

**Authors:** Li-Tao Guo, Anastasiya Grinko, Sara Olson, Alexander M. Leipold, Brenton Graveley, Antoine-Emmanuel Saliba, Anna Marie Pyle

**Affiliations:** 1 Department of Molecular, Cellular, and Developmental Biology, Yale University, New Haven, Connecticut 06520, USA; 2 Helmholtz Institute for RNA-based Infection Research (HIRI), Helmholtz-Centre for Infection Research (HZI), 97080 Würzburg, Germany; 3 Genetics and Genome Sciences, University of Connecticut Health, Farmington, Connecticut 06030, USA; 4 University of Würzburg, Faculty of Medicine, Institute of Molecular Infection Biology (IMIB), 97070 Würzburg, Germany; 5 Department of Chemistry, Yale University, New Haven, Connecticut 06520, USA; 6 Howard Hughes Medical Institute, Chevy Chase, Maryland 20815, USA

**Keywords:** MarathonRT, UltraMarathonRT, reverse transcriptase, template switching, RNA-seq, Smart-seq

## Abstract

End-to-end RNA-sequencing methods that capture 5′-sequence content without cumbersome library manipulations are of great interest, particularly for analysis of long RNAs. While template-switching methods have been developed for RNA sequencing by distributive short-read RTs, such as the MMLV RTs used in SMART-Seq methods, they have not been adapted to leverage the power of ultraprocessive RTs, such as those derived from group II introns. To facilitate this transition, we dissected the individual processes that guide the enzymatic specificity and efficiency of the multistep template-switching reaction carried out by RTs, in this case, by MarathonRT. Remarkably, this is the first study of its kind, for any RT. First, we characterized the nucleotide specificity of nontemplated addition (NTA) reaction that occurs when the RT extends past the RNA 5′-terminus. We then evaluated the binding specificity of specialized template-switching oligonucleotides, optimizing their sequences and chemical properties to guide efficient template-switching reaction. Having dissected and optimized these individual steps, we then unified them into a procedure for performing RNA sequencing with MarathonRT enzymes, using a well-characterized RNA reference set. The resulting reads span a six-log range in transcript concentration and accurately represent the input RNA identities in both length and composition. We also performed RNA-seq from total human RNA and poly(A)-enriched RNA, with short- and long-read sequencing demonstrating that MarathonRT enhances the discovery of unseen RNA molecules by conventional RT. Altogether, we have generated a new pipeline for rapid, accurate sequencing of complex RNA libraries containing mixtures of long RNA transcripts.

## INTRODUCTION

RNA sequencing (RNA-seq) has transformed our understanding of gene expression in studies of biological mechanism, evolution, and disease (Wang et al. 2009; Stark et al. 2019; Hong et al. 2020), but limitations in the way RNA-seq is experimentally performed restrict our understanding of transcriptome diversity and expression levels, particularly in the case of low-abundance RNAs (Mereu et al. 2020), structured RNAs (Boivin et al. 2020), or RNAs without known sequences at their 5′- or 3′-ends (Adiconis et al. 2018). Efficient amplification of RNA pools requires a “handle” at each terminus of a transcript so that cDNA synthesis, followed by second-strand synthesis, can be carried out. While mRNAs can readily be captured via primers to the 3′-poly(A) tail, subsequent second-strand synthesis is challenged by the lack of information on sequence at the 5′-end of the original RNA transcript. Methods involving 5′-cap capture (Takahashi et al. 2012), RNA cyclization (Chu et al. 2015), and adapter ligation (Ibrahim et al. 2021) have greatly improved outcomes, but they are not always efficient nor applicable to low-abundance transcripts or single-cell applications. The use of random hexamer primers has been highly successful, but the resulting short reads eliminate information on linkage among alternative spliced, edited or modified sites along a long natural transcript.

**FIGURE 1.**
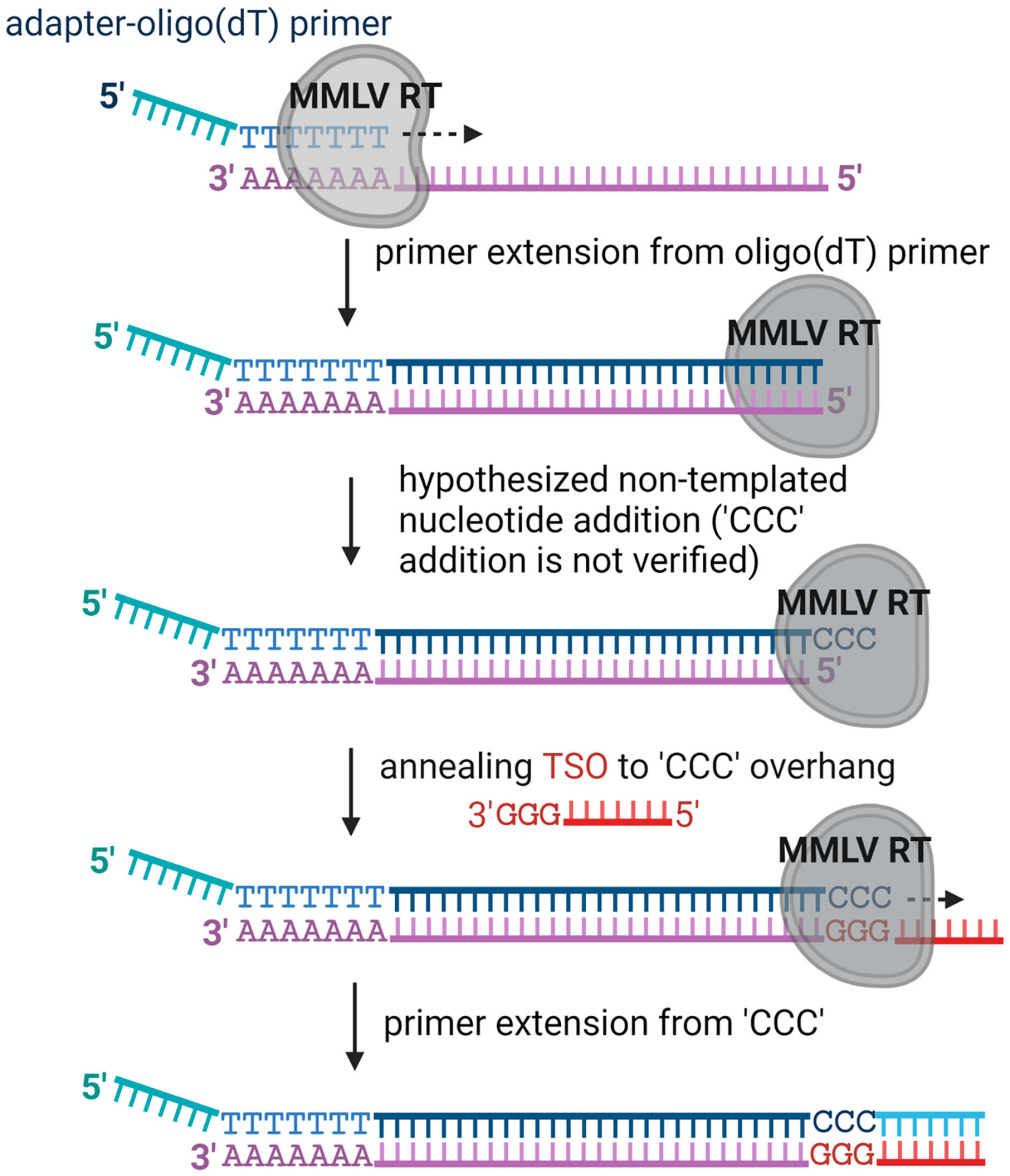
Schematic outline of cDNA synthesis on mRNA using the template switching activity of MMLV RTs. The outline depicts the steps, including oligo(dT) primer annealing, primer extension, nontemplated “CCC” addition, TSO annealing to the “CCC” overhang, and primer extension on TSO. The last three steps are known as template switching reactions, and nontemplated “CCC” addition and the subsequent TSO annealing step are based on hypotheses that have never been proven directly for MMLV RTs.

Given these issues with conventional amplification methodologies, there has been increasing attention to new kinds of end-to-end RNA-seq methods that faithfully capture the 5′-terminal sequences of RNA template pools (Adiconis et al. 2018). Among the most successful of these methods leverages the natural “template switching” behavior of many reverse transcriptase (RT) enzymes that are derived from Moloney murine leukemia virus (MMLV) RT (Islam et al. 2012; Batut and Gingeras 2013; Picelli et al. 2013). As outlined in Figure 1, full-length cDNA synthesis mediated by, for example, oligo(dT) primers are followed by cDNA 3′ adapter addition mediated by a multistep template-switching reaction of these RT enzymes. Despite the promise of this method, the actual reactions and steps involved in template switching have never been systematically characterized or optimized, for any RT enzyme. Template switching is not a single process, but a series of enzymatic RT side-reactions that ultimately result in the ability to amplify 5′-terminal sequences (Fig. 1). Template switching by RT enzymes is essential for retroviral recombination (Katz and Skalka 1990; Kulpa et al. 1997) and, in the case of retroelement RTs, it may facilitate target-primed reverse transcription (TPRT) required for full integration into host genomes (Bibillo and Eickbush 2002, 2004). Template-switching results from the fact that, in addition to RNA-dependent DNA polymerase activity, RT enzymes can carry out two other activities: (i) RTs can have terminal transferase activity, resulting in the nontemplated nucleotide addition (NTA) of additional nucleotides to the 3′-end of cDNA products. The number and sequence of nontemplated nucleotides are highly variable and depend on the specific type of RT enzyme and (ii) Certain RTs can jump from one template strand to another RNA strand that is in the immediate neighborhood, where they pick up and resume cDNA synthesis (template switching). The combination of these three capabilities (RT, NTA addition, and template switching) has been leveraged to create sequencing methods that can be used without prior knowledge of the RNA-5′-end sequence.

Despite the promise of template-switching RNA-seq pipelines, the methods are limited by the fact that they use RTs derived from MMLV (such as SuperScript II [SSII] or SSIV) (Picelli et al. 2013; Hahaut et al. 2022). This class of enzymes has inherently low processivity, causing them to stop frequently before reaching the end of RNA templates, and to dissociate upon encounter with stable secondary structures in the RNA template (Harrison et al. 1998; Guo et al. 2022). In addition, MMLV RTs often switch templates in the middle of an RNA (Kulpa et al. 1997; Basu et al. 2008), during pausing events, which leads to truncated cDNA. Until now, template-switching methods have never been built around ultraprocessive RT enzymes that copy kilobase RNAs from end-to-end, and thereby report the full transcriptomic diversity of individual transcripts (such as discrete alternative splice and editing populations). A second problem with MMLV-based template-switching applications is that the individual NTA and switching reactions have not been characterized in detail, making it difficult to optimize them and design programmable sequencing systems. For example, switching efficiency is strongly impacted by the sequence and modification status of RNA template 5′ nt (Wulf et al. 2019). Taken together, these shortcomings of MMLV-based template-switching protocols prevent the generation of sequencing libraries that truly represent the abundance and diversity of natural transcriptomes.

To address these issues and to provide a new resource for expanding the scope of RNA-seq, we characterized and optimized a template-switching pipeline using a robust, ultraprocessive RT. Specifically, we chose to leverage the properties of an enzyme known as MarathonRT (MRT), a group II intron RT with well-characterized behavior and optimized properties (Zhao et al. 2018; Guo et al. 2020,^2022^) and broad implementation in methods ranging from sequencing to RNA structure probing (Ju et al. 2019; Huston et al. 2021; Ravindra et al. 2021; Tavares et al. 2021; Yamagami et al. 2022; Araujo Tavares et al. 2023; Bohn et al. 2023; Ju et al. 2023; Mitchell et al. 2023; Patel et al. 2023; Pekarek et al. 2023; Scheepbouwer et al. 2023). Rather than plugging MRT into existing workflows, we first wanted to ensure that the MRT pipeline was fully optimized and appropriately programmed. We therefore accompanied optimization with a comprehensive analysis of the individual steps that occur during template switching by MRT, establishing its sequence specificity and oligonucleotide cofactor requirements. Surprisingly, such a mechanistic characterization of RT template switching represents the first of its kind, as MMLV systems were never systematically optimized or explored prior to implementation. We show that MRT has repetitive NTA additions, and a strict sequence bias, enabling efficient annealing to appropriately programmed TSOs. We then derived a set of optimized template-switching protocols and then deployed them to sequence a standardized library of low-abundance RNA transcripts, resulting in a robust pipeline applicable for whole transcriptome analysis. This analysis reveals greater diversity in read composition and length than previously observed with retroviral RTs.

## RESULTS

### Characterizing nontemplated nucleotide addition activity of MarathonRT

NTA catalyzed by the terminal transferase activity of an RT enzyme, is the first step in the process of template switching during RNA-seq. The reaction efficiency, nucleobase identity, and length of NTA products have a profound impact on template switching specificity and efficiency. As a first step in using MRT for template-switching sequencing, we performed a systematic study on the NTA activity of the enzyme in order to determine its efficiency and nucleotide preference.

**FIGURE 2.**
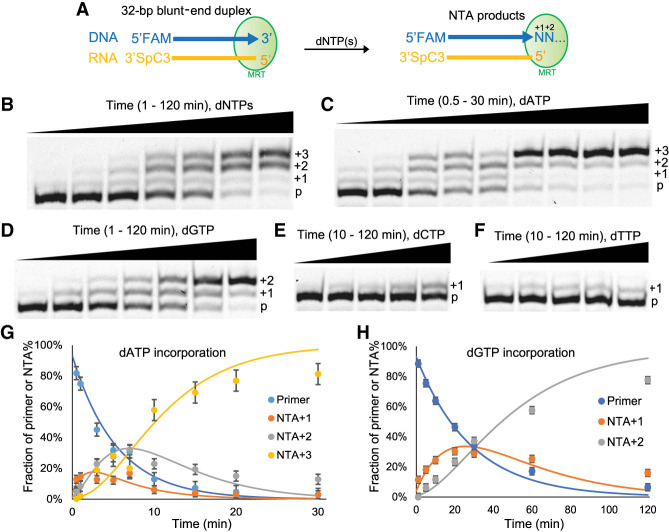
Examining the NTA activity and nucleotide preference of MarathonRT. (*A*) Outline of the experimental design for the NTA assay. A DNA/RNA blunt end duplex serves as the substrate, and the arrow of the DNA strand denotes the direction of NTA. The potential NTA products are described as different numbers of nucleotide additions as shown in the figure. (*B*) A representative gel figure showing the separation of NTA products of 1, 2, and 3 nucleotide additions (+1, +2, and +3) from the DNA primer (p) on a high-resolution polyacrylamide gel. An equimolar dNTP mix was used in this reaction. (*C*), (*D*), (*E*), and (*F*) Representative gel figures showing the separated NTA products from the DNA primer when using individual dATP, dGTP, dCTP, and dTTP in the reaction, respectively. (*G*) Regression modeling the production of one, two, and three adenosine additions to calculate the rate constant for each NTA product. (*H*) Regression modeling the production of one and two guanosine additions to calculate the rate constant for each of them. The curves were generated from the averaged result of three repeats for each reaction.

To focus specifically on the NTA activity of MRT, we designed a new experimental system. Specifically, we prepared a 32-bp blunt-end DNA/RNA duplex by annealing an oligo(dT) to an oligo(rA) (FAM_dT30GC and GCrA30, respectively, Supplemental Table 1), which serves as the substrate for MRT terminal transferase activity. The reaction was carried out by incubating MRT with the hybrid duplex and an equimolar dNTP mix in a previously reported reaction buffer that contains 50 mM Tris-HCl pH 8.3, 200 mM KCl, 2 mM MgCl_2_, 5 mM DTT, and 20% glycerol (Fig. 2A; Guo et al. 2020). FAM labeling at the 5′-end of the DNA strand facilitated high-resolution visualization and quantification of NTA products via polyacrylamide gel electrophoresis. To ensure that any terminal transferase activity would result in specific addition of dNTPs to the 3′OH of the DNA strand, the 3′OH of the RNA strand was chemically blocked (Supplemental Table 1).

Electrophoretic analysis of the NTA products revealed progressive addition of up to three nucleotides (Fig. 2B). The product band representing addition of one nucleotide was much weaker than the band representing 2 nucleotide additions across the time course, suggesting that the second nucleotide addition is fast enough to deplete the initial single-addition product. As NTA reactions for the first and second nucleotide nearly reached completion, a product representing addition of the third nucleotide appeared shortly after addition of the second nucleotide, indicating high efficiency for the third nucleotide addition (Fig. 2B). This NTA behavior diverges drastically from that of TGIRT, a thermostable group II intron-encoded RT, for which addition of the first nucleotide is nearly 10 times faster than the second nucleotide addition, and the third nucleotide addition is the slowest (Lentzsch et al. 2019). We used the same hybrid duplex to test NTA activity of TGIRT. In that case, we detected only 1 nt addition to the 5′-end of the DNA strand, and the reaction did not reach completion (Supplemental Fig. S1), which is likely due to the reduced stability of the oligo(dT)/(rA) duplex at 60°C, the optimal reaction temperature of TGIRT.

We then proceeded to characterize the nucleotide selectivity of the MRT terminal transferase. To this end, rather than using a dNTP mix, we examined MRT NTA activity in reactions containing individual dNTPs, quantifying the corresponding efficiency of the NTA reaction. Surprisingly, MRT efficiently incorporates dATP (Fig. 2C), appearing to vastly prefer it relative to the other three triphosphate nucleotides (Fig. 2D–F). Consecutive NTA of three nucleotides was only observed with dATP. Reaction with dGTP addition is much slower, and only two Gs can be incorporated (Fig. 2D). Reactions with dCTP and dTTP incorporation are very slow, and only a single nucleotide is partially incorporated after 2 h (Fig. 2E,F). A very faint +2 product is detectable for dTTP at late time points. Quantitation of the data enabled us to determine rate constants *k*_obs_ for the first and second adenosine addition, with values of 0.19 min^−1^ and 0.61 min^−1^, respectively, which are five and 15 times faster than the corresponding guanosine addition (Fig. 2G,H; Table 1). The third adenosine addition is also very efficient, with a rate constant of 0.20 min^−1^ and amplitude of 86% (Table 1). Given the strong preference for dATP, we concluded that, even in NTA reactions containing an equimolar dNTP mix (Fig. 2B), a triple-A NTA overhang is likely to dominate the 3 nt NTA products.

**TABLE 1.** The *k*_obs_ values and amplitude parameters for MarathonRT NTA activity with dATP and dGTP in the standard RT buffer

Identity of NTA nucleotide	No. of NTA nucleotide	*k*_obs_ (min^−1^)	Amplitude
dATP	1	0.19 ± 0.02	96%
	2	0.61 ± 0.18	96%
	3	0.20 ± 0.03	86%
dGTP	1	0.035 ± 0.008	94%
	2	0.040 ± 0.015	80%

The parameters were calculated from the average values of three independent experiments along with standard errors.

### Determination of MRT template-switching behavior

**FIGURE 3.**
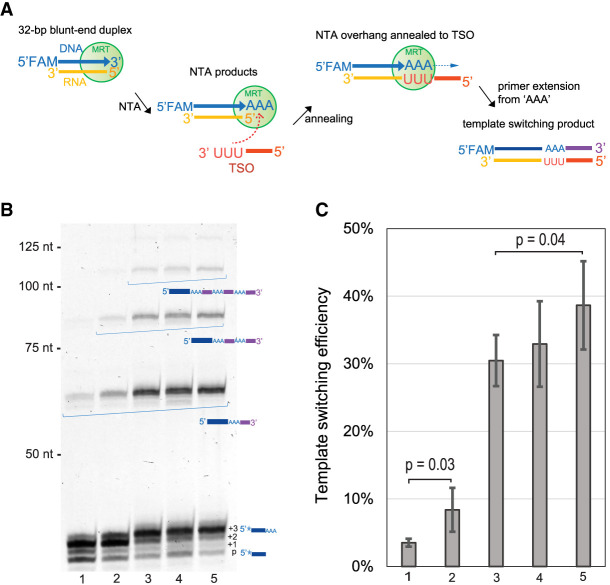
Sequential optimization of template switching activity of MarathonRT. (*A*) Outline of the experimental design to monitor the template-switching activity. The figure describes each step involved in the template-switching reaction of MarathonRT, including nontemplated “AAA” addition, annealing between the “AAA” overhang and 3′-UUU of an RNA TSO, and primer extension continuing on the TSO. (*B*) A gel figure showing the template switching products of MarathonRT under representative reaction conditions, including the original RT buffer with an equimolar mix of 0.5 mM dATP, dCTP, dGTP, and dTTP (lane *1*), the same reaction buffer as lane *1* but using a dNTP mix containing 0.8 mM dATP and 0.4 mM dCTP, dGTP, and dTTP (lane *2*), the same reaction condition as lane *2* with additional 12% PEG4000 (lane *3*), reduced KCl concentration from 200 mM to 150 mM compared to lane *3* (lane *4*), and further reduced KCl concentration to 100 mM (lane *5*). (*C*) Quantification of template switching efficiency for each condition in (*B*). Each reaction was repeated three times with mean and standard deviation shown in the figures. *P*-values were calculated using unpaired one-tailed Student's *t*-test.

Having investigated the nucleotide preference of MRT in NTA reactions, we set out to determine whether the enzyme can switch template strands and catalyze primer extension of an oligonucleotide template annealed to nontemplated nucleotides at the 3′ terminus of cDNA products (the template-switching oligonucleotide, or TSO). Given that MRT appends a triple-A overhang to cDNA products, even using a dNTP mix (Fig. 2B), we designed an RNA acceptor oligo containing 3′-r(UUU) as the TSO (MRT_TSO_UUU, Supplemental Table 1). We hypothesized that base-pairing between the 3′-r(UUU) of this TSO and the triple-A overhang will facilitate RT switching to the TSO, followed by primer extension (Fig. 3A). To examine the hypothesis, we combined the blunt-end DNA/RNA “substrate” duplex used in the previous NTA addition experiments (Fig. 2A) with the TSO (MRT_TSO_UUU, Supplemental Table 1), MRT and an equimolar dNTP mix for 1 h at 42°C in the same reaction buffer as that for NTA reactions (Fig. 3A). Electrophoretic analysis of the products confirmed that a template-switched primer extension reaction occurs with low efficiency (∼4%, Fig. 3B, lane 1; Fig. 3C, bar 1). We then used several strategies to improve the template-switching efficiency, using a strategy of sequential optimization.

First, instead of using an equimolar dNTP mix (containing 0.5 mM of each), we examined a biased dNTP mix, containing 0.8 mM dATP and 0.4 mM of the other three dNTPs. We expected that the higher dATP concentration might favor triple-A overhang production during the NTA reaction, which may in turn increase template-switching efficiency. Indeed, the biased dNTP mix increased efficiency (8%, Fig. 3B, lane 2 and 3C, bar 2). During the second round of optimization, we examined the effect of PEG4000 on the template-switching efficiency, since crowding agents have been demonstrated to improve cDNA yield in template-switching reactions by MMLV RTs (Bagnoli et al. 2018; Hagemann-Jensen et al. 2020). Indeed, the addition of 12% PEG4000 increased the template-switching efficiency to 30% (Fig. 3B, lane 3 and 3C, bar 3). In addition, the yield of trinucleotide NTA product is much higher in the presence of PEG4000, which may improve production of the triple-A overhang and/or subsequent template switching. Last, we examined the effect of the KCl concentration on template switching because it has been shown that lower salt concentrations favor template switching by TGIRT (Lentzsch et al. 2019). As in that case, lowering the KCl concentration from 200 mM in the original buffer (Fig. 3B, lane 3) to 150 mM (Fig. 3B, lane 4) or 100 mM (Fig. 3B, lane 5) moderately increases template-switching efficiency to 33% (Fig. 3C, bar 4) and 39% (Fig. 3C, lane 5), respectively. Based on these results, we finalized the optimal template-switching conditions for MRT: a biased dNTP mix (0.8 mM dATP and 0.4 mM of the other three dNTPs), 1 µM TSO and an optimized reaction buffer containing 50 mM Tris-HCl pH 8.3, 100 mM KCl, 2 mM MgCl_2_, 5 mM DTT, 20% glycerol, and 12% PEG4000. Time course experiments show that template switching in the optimized buffer reaches a plateau in 30 min (Supplemental Fig. S2). We noticed that, while MRT template switching proceeds efficiently in the optimized conditions, long TSO concatemers can be generated by a cycled template-switching reaction (Fig. 3B). During RNA-seq library preparation, such TSO concatemers extend the cDNA and can take over a large fraction of the sequencing reads. This was remedied by the adaptation of the TSO for RNA-seq applications (see section below on the RNA-seq pipeline).

### Establishing the specificity of TSO annealing during MarathonRT template switching

**FIGURE 4.**
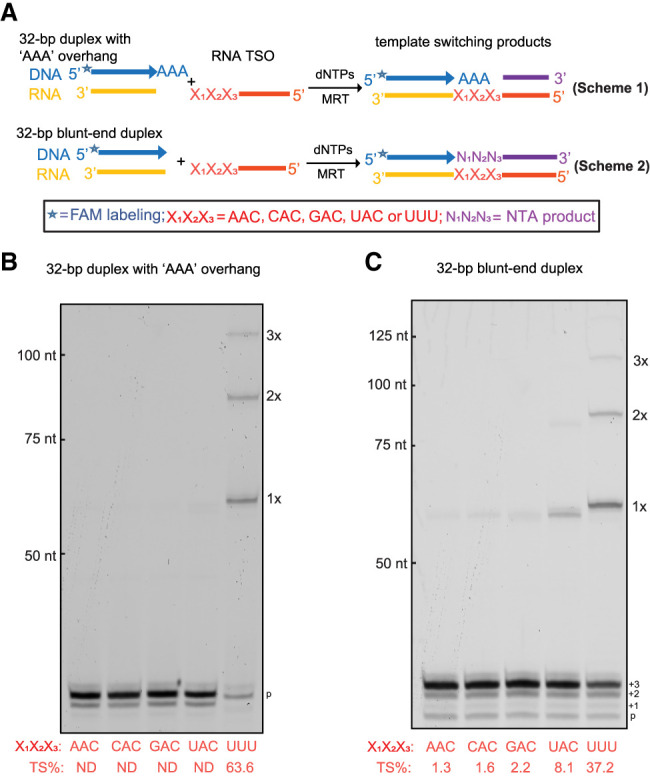
Examining the TSO annealing specificity and its relationship with template switching efficiency. (*A*) Outline of the experimental design that uses two sets of template switching reactions. As shown in the figure, in the first set of reactions (Scheme 1), a DNA/RNA duplex with 3′-d(AAA) overhang was used in combination with RNA TSOs of varying 3′ nt sequences. In the second set (Scheme 2), a blunt-ended DNA/RNA duplex was used in combination with the same set of TSOs. (*B*) Electrophoretic analysis of the template-switching products generated using the DNA/RNA duplex with 3′-d(AAA) overhang and (*C*) the blunt-ended DNA/RNA duplex. The template switching efficiency (TS%) is the average of three repeated experiments for each reaction shown in the figures.

Although it is commonly assumed that TSOs interact with NTA addition products via base-pairing (Zhu et al. 2001; Lentzsch et al. 2019), it has never actually been established experimentally for any RT enzyme through systematic mutational analysis. It is likely that many RT enzymes do not require strict base-pairing between TSOs and parent cDNA strands (Wulf et al. 2019). We therefore set out to determine the sequence specificity of MRT template switching and to establish whether it is mediated by Watson–Crick base-pairing between the NTA overhang and TSO 3′ nucleotides. To that end, we created a DNA/RNA duplex with a “d(AAA)” overhang by annealing a FAM-labeled DNA oligo FAM_dT30GCAAA with the RNA oligo GCrA30 (Fig. 4A, scheme 1; Supplemental Table 1) and designed four new TSOs with different 3′-end sequences (MRT_TSO_CAA, MRT_TSO_CAC, MRT_TSO_CAG and MRT_TSO_CAU, respectively, Supplemental Table 1 and Fig. 4A, scheme 1). None of the four TSOs can form base pairs with the “d(AAA)” overhang. Template-switching reactions were carried out by incubating the sticky-ended DNA/RNA duplex with each of the four TSOs or the TSO with 3′-r(UUU), respectively, under the optimized reaction condition (Fig. 4A, scheme 1). The result confirmed that template switching occurs only with the TSO containing 3′-r(UUU) (Fig. 4B) that can anneal to the “d(AAA)” overhang, and the efficiency reaches 63.6%. We also tested a DNA/RNA duplex with a single “dG” overhang (FAM_dT30GCG and GCrA30, Supplemental Table 1), and used this duplex to perform template-switching reactions with each of the five RNA TSOs. The result confirmed again that base-pairing between the single “dG” overhang and the 3′-end nucleotide of TSO (X_1_ position shown in Supplemental Fig. S3A) is required for template switching, and even a weak G:U base pair can mediate template switching though at a much lower efficiency (Supplemental Fig. S3B). The result also suggested that base pair strength dictates the template-switching efficiency.

### Determining template-switching specificity in mixed dNTP samples

Although dATP is most efficiently incorporated in NTA reactions, MRT does incorporate the other three dNTPs (Fig. 2D– F). Here, we set out to evaluate the relative abundance of NTA products generated from all four dNTPs when using the biased dNTP mix, which is achieved by exploiting the template-switching mechanism of MRT. To this end, we incubated the blunt-ended DNA/RNA duplex as used in the above experiments with each of the four newly designed TSOs (MRT_TSO_CAA, MRT_TSO_CAC, MRT_TSO_CAG, and MRT_TSO_CAU in Supplemental Table 1 and Fig. 4A, scheme 2), respectively. The 3′-end nucleotides of the four TSOs differ in identity. The template-switching efficiency with each TSO reflected the incorporation efficiency of the first NTA nucleotide that is complementary to the 3′-end nucleotide of the TSO. The TSO with 3′-r(UUU) was also included for comparison. The result showed that template switching to the TSOs ending with 3′-rA, -rC, and -rG is only 2% or less (Fig. 4C), suggesting that the incorporation of dTTP, dGTP and dCTP in the NTA reaction is minimal. In contrast, the efficiency of template switching to the TSO ending with 3′-rU is much higher (8%), agreeing well with the fact that dATP incorporation in NTA reactions is most efficient. However, template-switching efficiency remains most efficient when using a TSO with 3′-r(UUU) (Fig. 4C), suggesting that triple-A addition is faster than template-switching reaction mediated by the first adenosine addition and agreeing well with the above hypothesis that a triple-A overhang dominates the NTA products.

### The influence of the RNA template 5′-terminus on template-switching efficiency

During template-switching reactions, the RNA template is juxtaposed to the TSO within the active site of a group II intron-encoded RT (Lentzsch et al. 2021), where the 5′-end of the RNA template is next to the 3′-end of the TSO. The nucleotide identity and modification status of the RNA template 5′-end have been shown to strongly impact the template-switching efficiency of MMLV RTs (Wulf et al. 2019; Wulf et al. 2022), which biases the selection of RNA templates with the preferred 5′-nucleobase and 5′-modifications. Given these findings, we examined the effects of 5′-end nucleotide identity (including all four bases) and 5′-end modification status (including 5′ hydroxyl, 5′ triphosphate and 5′ m^7^G cap) of RNA templates on template-switching efficiency by MRT.

**FIGURE 5.**
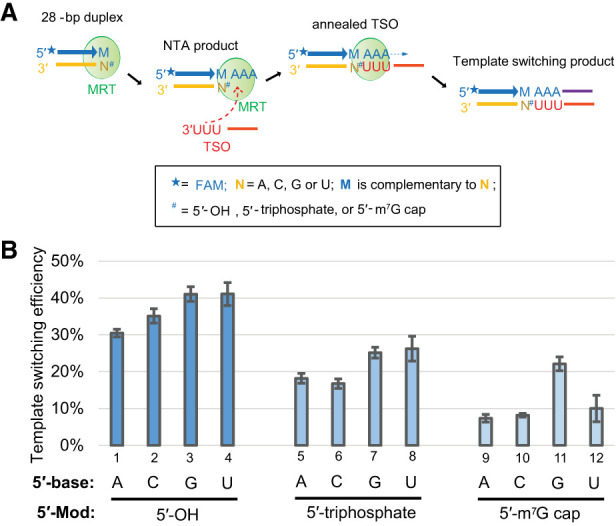
Evaluating the impact of RNA template 5′-nucleobase identity and 5′-modification on MarathonRT template switching efficiency. (*A*) Outline of the experimental design to analyze the impact of RNA template 5′-nucleobase identity and 5′-modifications on template-switching efficiency. The RNA template 5′-nucleobase identity and 5′-modifications are described in the legend of the figure. (*B*) Quantification of the template-switching efficiency using the RNA templates with different 5′-nucleobase identity and 5′-modifications. Each reaction was repeated three times with mean and standard deviation shown in the figures.

To this end, we designed three sets of RNA oligonucleotides that can serve as RNA templates for the template-switching reaction. Within each set, there are four RNA oligonucleotides that differ in the 5′-end nucleotide and share the same 5′-end modification (see Supplemental Table 1 for the oligonucleotide sequences of AGCrA25, CGCrA25, GGCrA25, and UGCrA25 that contain 5′ triphosphate, and see Materials and Methods for the generation of the other two sets that contain the 5′ hydroxyl and 5′ m^7^G cap, respectively). Each RNA was annealed to a FAM-labeled primer to make a blunt-ended DNA/RNA duplex that can undergo the template-switching reaction using the 3′-r(UUU) TSO (Fig. 5A). We find that the identity of the RNA template 5′ nt has minimal impact on MRT template-switching efficiency when RNA templates are terminated with a 5′ OH or 5′ triphosphate (Fig. 5B). For RNA templates terminating with a 5′ m^7^G cap, the template-switching efficiency is mildly biased toward RNA templates ending in 5′ G. The data confirm that modification at the RNA 5′-end does affect template-switching efficiency: larger 5′ chemical groups (m^7^G > triphosphate > hydroxyl in size) correlate with a reduction in efficiency, with average efficiencies of around 35%, 20%, and 10% for the RNA templates bearing a 5′ hydroxyl, 5′ triphosphate and 5′ m^7^G cap, respectively (Fig. 5B). That said, template-switching efficiency is sufficiently high in the presence of 5′ m^7^G for preparation of sequencing libraries.

### Establishing an RNA-seq pipeline using MarathonRT template switching

Given that MRT template switching is efficient and proceeds with a high level of specificity via the 3′-AAA overhang, we reasoned that it could be used to simplify RNA-seq pipelines, much like Smart-seq (Picelli et al. 2013), thereby providing sensitive end-to-end coverage of RNA transcripts of any length during transcriptome profiling. To that end, we sought to establish a full-length RNA-seq approach by integrating the MRT template-switching reaction into next-generation sequencing (NGS) library preparation. To benchmark the performance of the libraries, we chose SIRV-Set 3 RNA spike-in variants (Lexogen) as the templates and used only 0.1 ng as the input. SIRV-Set 3 is a set of polyadenylated synthetic RNAs that contain 161 transcripts that include the 92 transcripts of ERCC (External RNA Controls Consortium) spike-in control mix and 69 SIRV isoforms. The 92 ERCC transcripts are designed to be 250–2000 nucleotides in length, mimicking natural eukaryotic mRNAs with concentrations spanning six orders of magnitude; the SIRV isoforms consist of 69 artificial transcript variants that mimic the splicing characteristics of seven human model gene loci, with length ranging from 200 to 2500 nucleotides. To prepare sequencing libraries of this mixture, the set of SIRV-Set 3 RNA spike-in variants was first converted to full-length cDNA using oligo(dT) priming and template switching by MRT (as above, see Materials and Methods), followed by PCR preamplification of cDNA. We then used the amplified cDNA to construct standard Illumina sequencing libraries using Tn5-mediated “tagmentation” using the Nextera technology (Tn5) (see Materials and Methods for details).

During RNA-seq library preparation, we encountered two issues with the TSO and one issue with the template-switching reaction buffer. First, the TSO can be autonomously reverse-transcribed by the RT primer via a single base pair. To prevent this non-specific primer and TSO amplification, we optimized their nucleotide sequences (MRT_dT18_opt and MRT_TSO_opt, Supplemental Table 1) by using C and T only to remove any potential base pairs between them. Second, we also found that multiple copies of TSO were concatenated by cycled template-switching reactions (Fig. 3B) and the TSO concatemers dominate the sequencing reads. To reduce TSO concatenation, we first used a DNA TSO in place of an RNA TSO to carry out template-switching reactions, and we hoped to reduce TSO concatenation by lowering overall template-switching efficiency. In addition, we attached one or more large chemical groups to the 5′-OH of TSO to further reduce TSO concatenation (Supplemental Fig. S4). We examined four types of 5′-modifications, including five consecutive abasic sites (MRT_TSO_5ab, Supplemental Table 1), three consecutive abasic sites (MRT_TSO_3ab, Supplemental Table 1), a trebler (MRT_TSO_trebler, Supplemental Table 1), and a trityl (MRT_TSO_trityl, Supplemental Table 1). By using any of the 5′-modified DNA TSOs with optimized sequence to prepare a sequencing library, 95% of sequencing reads were mapped to the SIRV-Set 3 templates; as a comparison, the mapping rate was only 2% when using the TSO without a 5′-modification to prepare a library (Supplemental Table 2). We also examined the preamplified full-length cDNA libraries using Agilent BioAnalyzer 2100. The BioAnalyzer gel image shows that the library generated by the TSO without a 5′-modification exhibits a ladder pattern (Supplemental Fig. 5A), suggesting that this library is dominated by different lengths of TSO concatamers, which explains the low mapping rate of sequencing reads from this library (Supplemental Table 2). The ladder pattern is absent for the other four preamplified full-length cDNA libraries generated using TSOs containing a bulky 5′-modification (Supplemental Fig. S5A), suggesting that the 5′ modifications successfully prevent concatenation of TSO during the template-switching reaction.

An additional issue we identified is that the resulting cDNA libraries contain only short DNA products, of which nearly all are below 600 bp (Supplemental Fig. S5A), thereby failing to represent the length distribution of SIRV-Set3 templates. The most abundant transcripts in SIRV-Set3 RNA are ∼1050 nt in length and the second most abundant transcripts are ∼ 500 nt, which are absent in all the above full-length cDNA libraries (Supplemental Fig. S5A). These findings suggested that the preamplified cDNA libraries do not cover the entire length of the SIRV-Set3 RNA transcripts. We hypothesized that the reaction condition that had been specifically optimized for the template-switching reaction by MRT (Fig. 3A, lane 5) may not be optimal for primer extension, thereby leading to truncated cDNA products. To correct this, we used a sequential two-step process to separate the primer extension reaction from the template-switching reaction, thereby allowing us to use the optimal reaction conditions for primer extension and template switching, respectively, in order to maximize the performance of MRT (see Materials and Methods). For these experiments, we used the TSO with five abasic sites at the 5′-terminus during the template-switching reaction (MRT_TSO_5ab). Under these conditions, the cDNA library successfully captures the two major groups of RNA containing transcripts of ∼500 nt and ∼1050 nt in length (Supplemental Fig. S5B).

A remaining concern, however, was that DNA products of ∼500 nt appeared to be more abundant than those of ∼1050 nt (Supplemental Fig. S5B), suggesting that MRT copied the shorter RNA transcripts more efficiently, which is uncharacteristic for this enzyme. Given the very low RNA input (0.1 ng) used in the reverse transcription, we hypothesized that the secondary RNA-binding sites on MRT might interact nonspecifically with the pool of RNA templates, thereby interfering with normal RT function (Zhao et al. 2018). To address this issue, we repeated the experiment with an improved version of MRT, previously designated mut1 (Zhao et al. 2018). This variant contains mutations that reduce nonproductive enzyme–RNA interactions, which might impact full-length cDNA library preparation. The resulting data show that mut1 efficiently copies the long RNA transcripts (>1000 nt) and generates a full-length cDNA library that faithfully represents the SIRV-Set3 templates, which outperforms the wild-type MRT (Supplemental Fig. S5B). For this reason, we renamed mut1 as UltraMarathonRT (uMRT), because of its improved performance.

### Evaluating library preparation quality using ERCC reads

**FIGURE 6.**
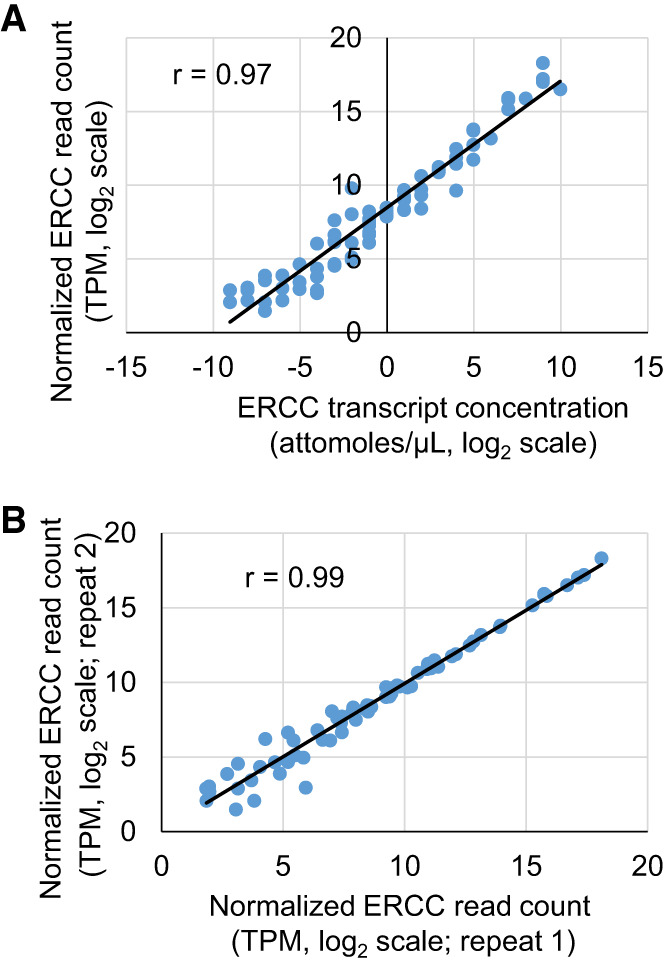
Examining the performance of ERCC RNA-seq library prepared using the template-switching reaction of uMRT. (*A*) Linear regression to examine the correlation between ERCC RNA transcript concentration (*x*-axis) used in library preparation and the normalized read count (TPM) (*y*-axis) calculated from the sequencing reads. The TPM values and ERCC transcript concentrations were log2 transformed. (*B*) Examining the reproducibility of RNA-seq experiment by comparing the normalized ERCC read count (log2 transformed TPM) of two repeated experiments.

Having successfully built SIRV-Set 3 RNA-seq libraries using an optimized primer, a specialized TSO, and a two-step procedure with uMRT, we proceeded to analyze the resulting sequencing data to quantify the abundance of the ERCC transcripts and to compare these with the actual input. As described above, the sequencing data used in this analysis were generated from the library prepared using uMRT and TSO MRT_TSO_5ab. In this data set, the abundance of a transcript represents its expression level, which can be quantified by the number of reads that map to the transcript sequence. Due to the nature of shotgun sequencing, longer RNA transcripts accumulate more reads. Therefore, the transcripts per million (TPM) value, a transcript length normalized read count (Wagner et al. 2012; Conesa et al. 2016), was calculated for each ERCC transcript and plotted against its known RNA input concentration. As shown in Figure 6A, the sequencing data displayed excellent linear performance across the full dynamic range of the ERCC transcript concentrations, from the transcripts of low to high abundance, spanning six orders of magnitude. Importantly, the data do not show saturation for the most abundant transcripts. For the transcripts with the lowest concentrations, there are only one or two pairs of reads among the ∼ 200,000 reads aligned to the ERCC templates, suggesting that the lower detection limit of quantitative measurement is limited by sequencing depth. Importantly, overall transcript quantification is highly reproducible (Fig. 6B).

### Evaluating uMRT performance in RNA-seq using total cellular RNA

Having confirmed the sequencing library quality using ERCC transcripts, we then performed RNA-seq experiments using universal human reference RNA (Novoradovskaya et al. 2004), which is commercially available (see Materials and Methods). Universal human reference RNA combines the total RNA from ten human cell lines, thereby representing a transcriptome with a highly complicated composition. It is therefore ideal for evaluating the performance of different RNA-seq pipelines and its wide availability ensures that anyone can perform comparative experiments on the same RNA source material. Sequencing libraries of two technical replicates were constructed using the template-switching reaction of uMRT, as performed for the SIRV library described above. To compare uMRT template-switching RNA-seq with a conventional methodology, sequencing libraries were also prepared using the template-switching reaction of SSII, the RT enzyme used in Smart-seq2, which is reported to display high sensitivity even with low RNA input (Picelli et al. 2013) and which is also used in the commercial TruSeq protocol from Illumina. On average, 30 million reads were acquired for each library.

Gene abundance determinations show equal reproducibility for uMRT (*r* = 0.88) and SSII (*r* = 0.88) based on the two technical replicates obtained for each RT enzyme (Supplemental Fig. S6A,B). These data indicate that uMRT performs at least as well as SSII when preparing a complex RNA-seq library from total RNA. Interestingly, the gene abundance correlation between the uMRT and SSII data sets is similar to the intra-replicate (uMRT Rep1 vs. SSII Rep1 and uMRT Rep2 vs. SSII Rep2) correlation (*r* = 0.83 for replicates 1 and *r* = 0.84 for replicates 2, respectively; Supplemental Fig. 6C,D). We observed that for both RTs, replicate 2 detects a higher number of TPMs for longer genes on average than replicate 1, possibly indicating a potential bias coming from an over- or under tagmentation in one of the replicates (Supplemental Fig. 6E,F).

**FIGURE 7.**
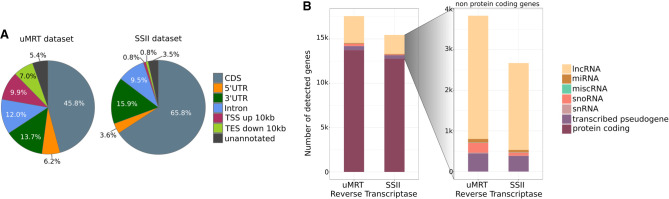
Comparison of gene detection by uMRT and SSII on universal human reference total RNA using Illumina short-read sequencing. (*A*) Pie charts depicting the proportional distribution of mapped sequencing reads across genomic features captured by uMRT (*left*) and SSII (*right*), respectively. Read assignment and mapping were both performed using the human reference GRCh38 (RefSeq GRCh38.p14, GCF_000001405.40, primary assembly) with STAR (v2.7.11b) and RSeQC (v5.0.1). Pie slices are colored according to genomic features. (*B*) Bar charts illustrating the mean number of genes detected (TPM > 0) between both replicates of uMRT and SSII, respectively, with reads downsampled to 20 million for every individual sample to account for sequencing depth. Colors indicate the gene biotype of the detected genes depicting the distribution across all detected biotypes (*left*) and zoomed in on non-protein-coding genes (*right*).

To assess the diversity of genomic features captured by both reverse transcriptases, we determined the fraction of sequence reads that were mapped to specific genomic features providing an overview of read distribution. Surprisingly, only 45.8% of the sequencing reads from the uMRT data set were mapped to annotated protein-coding sequences, compared to a corresponding fraction of 65.8% from the SSII data set (Fig. 7A). In contrast, the uMRT data set shows a more diverse distribution of mapped reads across all genomic features. While the fraction of reads assigned to intron regions, 5′UTRs, and 3′UTRs is similar between both replicates, there is an increase in reads that arise from sites upstream of transcription start sites (TSS) and downstream from transcription end sites (TES) in uMRT (9.9% for TSS and 7.0% to TES in uMRT compared to 0.8%, respectively, in SSII; Fig. 7A), potentially reflecting RNA structure in these transcript regions that SSII has difficulty traversing. Together, these data suggest that RNA-seq libraries prepared using uMRT enable a more diverse and unbiased interrogation of the transcriptome, which is expected based on previous studies (Zhao et al. 2018; Guo et al. 2022).

To account for any differences in sequencing depth between uMRT and SSII, we in silico downsampled the libraries to equal sequencing depths of 20 million reads and compared the number of detected genes. We observed that uMRT captures a mean of 17,775 annotated genes, whereas SSII only captures a mean of 15,526 annotated genes (Fig. 7B). Notably, uMRT captures more non-protein-coding transcripts, particularly lncRNAs (Fig. 7B). This observation emphasizes again that uMRT effectively captures a broader diversity of the transcriptome than SSII, rendering it particularly suitable for unbiased detection in RNA-seq.

### Evaluation of uMRT for long-read-based RNA-seq

To evaluate the full-length reverse transcription products of uMRT, we conducted Oxford Nanopore (ONT) long-read sequencing on mRNA obtained from the Huh 7.5 cell line after poly(A) enrichment. Similar to the analysis described above, we generated sequencing libraries of two technical replicates using the template-switching reactions of uMRT and compared these to the template-switching products of Maxima H minus RT (Thermo Fisher). The Maxima H minus RT enzyme was selected based on ONT's recommendation for library preparation, ensuring a robust benchmark for our study. After sequencing, we obtained an average of 2.5 million reads for each library.

Gene abundance correlations demonstrate excellent reproducibility for both uMRT (*r* = 0.98) and Maxima (*r* = 0.98), respectively (Supplemental Fig. S7A,B). Interestingly, intra-replicate correlations (uMRT replicate 1 vs. Maxima replicate 1 and uMRT replicate 2 vs. Maxima replicate 2) showed slightly lower correlations (*r* = 0.93, respectively, Supplemental Fig. S7C,D), indicating that while the two RTs identify the same set of core targets across replicates, they each recognize slightly different sets of transcripts.

**FIGURE 8.**
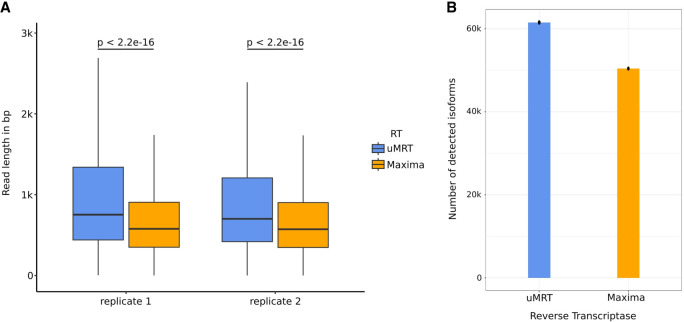
Sequencing read length and isoform detection comparison of uMRT and Maxima on poly(A) selected RNAs extracted from Huh7.5 cell line using ONT long-read sequencing. (*A*) Boxplots of sequencing read length in bp across replicate 1 and replicate 2 of uMRT and Maxima, respectively. Statistical significance was assessed using a two-sided Wilcoxon rank-sum test. Center line indicates the median, box limits indicate the upper and lower quantiles, and whiskers indicate the 1.5× interquartile range. (*B*) Bar charts depicting the mean number of isoforms detected (CPM > 0) between both replicates of uMRT and Maxima, respectively, with reads downsampled to 1.2 million for every individual sample to account for sequencing depth.

We compared the lengths of the captured sequencing reads between uMRT and Maxima. Both uMRT replicates exhibited higher mean read lengths (1110.9 bp and 971.8 bp for uMRT replicates 1 and 2, respectively) compared to Maxima (706.9 bp and 703.9 bp for Maxima replicates 1 and 2, respectively, Fig. 8A). These findings indicate that uMRT is more effective in capturing full-length sequences than conventional RTs, as expected given the known properties of this enzyme class.

Next, we assessed the ability of both RTs to identify known isoforms. We downsampled reads to equal sequencing depth (1.2 million reads) and found that uMRT and Maxima identified 61,514 and 50,410 isoforms, respectively (Fig. 8B). We repeated correlation analysis on the isoform level and found a better correlation of isoform abundance between uMRT replicates (*r* = 0.89, Supplemental Fig. S7E) compared to Maxima replicates (*r* = 0.85, Supplemental Fig. S7F), with correlations between RTs dropping substantially (*r* = 0.72, Supplemental Fig. S7G,H). Taken Together, these results suggest that uMRT exceeds the ability of Maxima to capture diverse isoforms, while maintaining comparable or even better reproducibility.

## DISCUSSION

Although many powerful transcriptome and epitranscriptome analysis pipelines are now built upon a foundation that requires template switching by RT enzymes, there is remarkably little known about the actual enzymatic steps that occur during cDNA generation via template switching (Fig. 1). In addition, a general framework for optimizing the specificity and efficiency of each step of this process has been lacking. RTs are complex multifunctional enzymes, each of which is capable of diverse mechanical behaviors and enzymatic activities. To fully leverage their power, and to better understand their biology, it is important to undertake comprehensive mechanistic analysis of any RT enzyme that is used as a tool enzyme in biotechnology. Here we dissect the individual enzymatic steps involved in template switching by the processive RT enzyme known as MRT (Figs. 2–4), which belongs to the structural class shared by group II intron and non-LTR retrotransposon RTs, which is architecturally distinct from retroviral RTs (such as HIV RT and MMLV RT) (Ding et al. 1998; Das and Georgiadis 2004; Zhao and Pyle 2016).

We first examined the initial reaction required for template-switching: the NTA activity of MRT (Fig. 2). Intriguingly, the NTA or terminal transferase activity of MRT is highly sequence and length-specific, resulting in the addition of three adenosines to the 3′-end of cDNA products. Kinetic analysis shows that after the first nucleotide is added, the next two rapidly follow, resulting in the homogeneous production of three nucleotide “sticky-ends” composed of three adenosines (Fig. 2C). This behavior contrasts with the apparent sequence and length specificity of other RT enzymes, as TGIRT adds 1 G or 1 A most efficiently (Lentzsch et al. 2019) and MMLV RT adds highly variable NTA products (Wulf et al. 2019), although MMLV RT and its variants are thought to add CCC during the NTA reaction (Zajac et al. 2013). This diversity of NTA specificity and length-dependence, even among RTs from the same structural family, suggests that NTA activity rapidly evolves in RT enzymes in response to certain selective pressures. From a practical standpoint, this finding demonstrates that RT enzymes are programmable components with great utility in RNA-seq, synthetic biology, and nanotechnology.

Recognition of the TSO was then examined. Upon consulting the literature, we were surprised to find that a systematic mutational examination of the requirement for base-pairing between TSO and NTA-extended cDNA had not been conducted, although it has long been assumed that base-pairing between the “sticky ends” of these two components is required for successful template switching (Zhu et al. 2001; Picelli et al. 2013). Given that MRT adds three A's, rather than a single nucleotide as in other systems, it was particularly important to determine if base-pairing specificity helps to drive the efficiency and precision of template switching by the enzyme, as this would underscore the programmability of MRT template-switching systems. To this end, we mutated both cDNAs and TSO sequences to evaluate the influence of mismatches in their pairing on subsequent template-switching efficiency. Indeed, we observed that perfect Watson–Crick pairing between the nontemplated nucleotides and the TSO greatly stimulates subsequent template switching by the RT, particularly under reaction conditions conducive to strengthening of base-pairing interactions (Fig. 4; Supplemental Fig. S3).

During the course of these experiments, it became immediately apparent that multiple-length products were being generated by successive addition of TSO extension products onto the same cDNA. This is because NTA addition can occur at each step, resulting in unproductive multiple addition of template-switched products, thereby reducing the signal to noise in downstream sequencing experiments. To block this process, we created a novel type of TSO that is designed to make it sterically “uncomfortable” for the polymerase to initiate a second time on TSO-cDNA products. Indeed, we showed that TSO's bearing bulky groups, such as polymers of abasic residues, or trebler moieties can prevent downstream concatenation and ensure that template switching occurs only one time (Supplemental Fig. S5).

Having independently examined the earliest step of the template-switching process (NTA addition), we then focused on optimization of the actual cDNA extension reaction by MRT, whereby the TSO becomes the template, the NTA-extended cDNA acts as the primer, and annealing between the 3′-end of TSO and the NTA overhang occurs in the active site of MRT. We found that this extension reaction is stimulated by PEG4000 and lower salt concentration (Fig. 3), which are different from the conditions for normal primer extension by MRT, as efficient primer extension by MRT requires a higher salt concentration (Guo et al. 2020). We reasoned that the 3′-end of TSO needs to enter the active site of MRT to anneal to the NTA overhang, and that a high salt concentration may interfere with the interaction between MRT and TSO and thus the annealing process. One aspect of the RNA/cDNA/TSO substrate that does differ from “normal” RT reactions is that the 5′-end of RNA molecules can contain modifications, such as caps and triphosphates that might be expected to inhibit extension of the NTA-modified cDNA. In addition, we wondered whether the base identity of the RNA 5′-nucleotide might influence efficiency of template switching. There is a small 5′-RNA base preference, and 5′-triphosphates show a small amount of inhibition. Importantly for mRNA sequencing, we find that 5′-caps are somewhat inhibitory, although the reaction still proceeds sufficiently well to facilitate library preparation. We expect that, when RNA input is very low, an RNA decapping step may be needed to reduce bias and enable efficient template switching.

With a functional template-switching procedure in place for MRT, we were able to prepare high-quality libraries for next-generation sequencing. Unlike MMLV RT, MRT or uMRT is not affected by the sequence, structure, or length of RNA transcripts during primer extension and its template-switching activity shows minimal bias toward the identity and modification status of the RNA template 5′ nucleotide. Because template switching occurs after uMRT reaches the ends of RNA transcripts, this enzyme is very useful to generate unbiased libraries from full-length RNA. As demonstrated in Figure 6A, the RNA-seq experiment based on the uMRT template-switching reaction accurately captures the abundance of input ERCC transcripts across a wide dynamic range. Moreover, uMRT expands the spectrum of RNA transcripts than can be captured by RNA-seq when using total RNA or poly(A) selected RNA as the input and sequencing using short or long reads (Figs. 6–8). In certain cases, the number of detected transcripts increases as well. For example, the number of lncRNA molecules captured by the uMRT is 30% greater than that for SSII. Furthermore, coverage uniformity is enhanced by uMRT, allowing one to increase reads that map to TSS and TES regions by a factor of 10 (Fig. 7A). Importantly, the sequencing experiments demonstrate high reproducibility (Fig. 6B, Supplemental Figs. 6A, 6G, 7A, 7E), thereby successfully fulfilling the intended objectives of standard RNA-seq experiments (Wang et al. 2009).

Given the utility of MRT and uMRT for generating long reads, it was important to conduct nanopore sequencing. Here, we show that the nanopore sequencing reads from the Maxima data sets are on average ∼300 nt shorter than those from the uMRT data sets (Fig. 8A). Benchmarking studies of ONT-based long-read RNA-seq applied on diverse cell lines and library preparation methods report mean read lengths spanning from 500 bp to 900 bp (Dong et al. 2023; Pardo-Palacios et al. 2024), which underscores the value of uMRT for characterization of long transcripts.

In the case described here, it is unlikely that primer extension on Huh 7.5 mRNA by Maxima is limited by stopping at stable RNA structures, since mRNAs are relatively unstructured and can be easily copied by most RT enzymes. It has been known that retroviral RTs, including MMLV RT and its variants, are distributive enzymes with inherent low processivity. They undergo frequent disassociation and reassociation during primer extension on a long RNA template, where they can switch to another template (such as a TSO) in the middle of the target RNA template during the pauses, thereby resulting in a shortened cDNA product, which agrees well with the previous studies (Kulpa et al. 1997; Basu et al. 2008).

Read length is the most critical factor influencing accurate isoform detection (Pardo-Palacios et al. 2024), and maximizing length is therefore a major goal for the development of RNA-seq pipelines. The uMRT read length advantage resulted in a 22% increase in isoform detection compared to Maxima, when starting from the same input material. We noticed substantial isoform drop-out when comparing different replicates for both uMRT and Maxima. Drop-outs emerged from lowly abundant isoforms combined with the relatively low sequencing depth. Indeed, benchmarking studies have identified sequencing depth as the main determinant for accurate isoform detection (Pardo-Palacios et al. 2024). Further studies will allow us to dissect how uMRT improves the assignment to transcript structural categories. While we have focused on the analysis of known, well-annotated isoforms, we expect uMRT to allow the discovery of unknown variants and to enhance the annotation of poorly annotated genomes. Finally, the choice of the pipeline for isoform annotation and quantification strongly influences the outcome of the analysis. Here, we focused on Bambu (Chen et al. 2023) because of its remarkable performance in capturing isoforms (Pardo-Palacios et al. 2024). It will be interesting to apply other pipelines in parallel to assess their ability to retrieve novel transcripts, especially when it comes to structured transcripts and/or transcripts arising from repetitive regions (Chen et al. 2023).

The template-switching activities of TGIRT and *Bombyx mori* R2 RT have also been successfully exploited to prepare RNA-seq libraries, but they require small RNAs or fragmentated long RNA as the input (Nottingham et al. 2016; Upton et al. 2021; Ferguson et al. 2023). In contrast, the template-switching activity of MRT is extremely useful as it works on long transcripts and can therefore be used to reflect a broader spectrum of transcriptome and epitranscriptome diversity during RNA-seq experiments. Its greatest impact will be on single-cell and spatial transcriptomics experiments due to simpler library preparation, and the fact that one obtains all the spliced and edited isoforms for each transcript in the pool. Normally, single-cell and spatial methods report transcript identity from short single reads that do not encompass the entire RNA. MRT makes it possible to obtain information on each intact transcript and its variation as a function of cell type, position, and growth condition, resulting in substantially more information on gene expression. In addition to alternative splicing and RNA editing diversity, epitranscriptome information can also be captured using MRT template-switching experiments that incorporate mutational profiling pipelines, such as those recently used to identify positions of RNA modifications, including all 2′-O-Methyl, N^7^-methyl-G and other common modifications (Araujo Tavares et al. 2023). Finally, implementation of a MRT template-switching pipeline will eliminate bias in the types of transcripts that are copied into cDNA. Using current protocols that use retroviral RTs such as SSIV, one does not detect RNAs that contain stable secondary structures or repeat sequences (Brooks et al. 1995; Malik et al. 2017; Guo et al. 2022), which eliminates a vast set of important transcripts. Because MRT remains bound to the template without dissociating, unwinding RNA structures in its path, MRT copies all transcripts regardless of their structural and sequence features (Zhao et al. 2018; Guo et al. 2022). This means that RNA-seq data generated using MRT will reveal new transcripts previously masked by the poor processivity of retroviral RTs (Boivin et al. 2020; Figs. 7 and 8), which will shed new light on genes implicated in neuromuscular disorders, cancer, and other riboregulated transcripts.

## MATERIALS AND METHODS

### Reagents

MRT, a group II intron-encoded RT derived from *Eubacterium rectale* (Zhao and Pyle 2016), and its variant uMRT (which is the same as mut1 in previously published work) (Zhao et al. 2018) were overexpressed in *E. coli* and purified to homogeneity as described previously (Guo et al. 2020). TGIRT is a thermostable group II intron-encoded RT from *Geobacillus stearothermophilus* (Mohr et al. 2013), purchased from InGex.com.

For the DNA and RNA oligos used in this work, detailed information was provided in Supplemental Table 1. The four FAM-labeled DNA primers, the RT primer (MRT_dT18_opt), the primers used to amplify cDNA (AmpPCR), and the DNA TSO with no 5′ modification (MRT_TSO_opt) were synthesized by Thermo Fisher. The four RNA oligos that contain 5′-triphosphates and the DNA TSOs containing 5′ modifications were synthesized in house and purified by 20% urea (7 M) denaturing polyacrylamide gel. All the other RNA oligoes were purchased from Integrated DNA Technologies (Coralville, IA) in RNase-free HPLC-purified form.

### NTA assays

To prepare the 32-bp blunt-end DNA/RNA starter duplex, 1 μL of 1 μM FAM-labeled DNA primer (FAM_dT30GC, Supplemental Table 1) and 1.2 μL of 1 μM complementary RNA oligo (GCrA30, Supplemental Table 1) were mixed, heated to 95°C for 45 sec, and then cooled down on ice. The reaction was set up by mixing the annealed starter duplex with 500 nM MRT or TGIRT, reaction buffer, and purified water (Millipore) to make a reaction volume of 9.5 μL. The reaction mixture was preincubated for 1 min at 42°C for MRT and at 60°C for TGIRT (ingex.com), and then initiated by adding 0.5 μL equimolar dNTP mix (10 mM each, NEB) or 0.5 μL unbalanced dNTP (16 mM dATP and 8 mM for the other three dNTPs). The reaction buffer for MRT contains 50 mm Tris-HCl, pH 8.3, 200 mm KCl, 2 mM MgCl_2_, 5 mM DTT, and 20% glycerol, which was optimized for efficient reverse transcription (Guo et al. 2020). For TGIRT, the reaction buffer contains 20 mM Tris-HCl, pH 7.5, 450 mM NaCl, 5 mM DTT, and 5 mM MgCl_2_ as suggested by the manufacturer. The reactions were incubated for the specified reaction times described in the text and then stopped by mixing with 2 μL 10% SDS. The reaction temperatures are 42°C for MRT and 60°C for TGIRT. The resulting NTA products were treated with 300 mM NaOH at 95°C for 5 min to hydrolyze the RNA strands, and then analyzed by electrophoresis on a 12% urea (7 M) denaturing polyacrylamide gel. The FAM-labeled gel was directly scanned by the Typhoon FLA 9500 scanner (GE Healthcare) under fluorescence channel. The gel images were quantified by ImageQuant TL 8.2 (GE Healthcare), and the resulting data for dATP and dGTP incorporation were analyzed by Kintek software.

### Template-switching assays

The template-switching reactions were carried out in the same way as NTA reactions except that an RNA or DNA TSO with the final concentration of 1 μM was included in each template-switching reaction. The template-switching products were analyzed by electrophoresis, visualized by the Typhoon FLA 9500 scanner, and quantified using ImageQuant TL 8.2 as described above. The overall template-switching efficiency was calculated using all the template-switching products, including the first and multiply switched products. The initial reaction buffer contains 50 mM Tris-HCl, pH 8.3, 200 mM KCl, 2 mM MgCl_2_, 5 mM DTT, and 20% glycerol, the optimal condition for reverse transcription (Guo et al. 2020). The optimized template-switching buffer contains 50 mM Tris-HCl, pH 8.3, 100 mM KCl, 2 mM MgCl_2_, 5 mM DTT, 20% glycerol, and 12% PEG4000.

### Removal of 5′-triphosphate from AGCrA25, CGCrA25, GGCrA25, and UGCrA25 to prepare RNA oligo templates containing 5′-OH

To remove the 5′-triphosphates from the 28-mer RNA oligos (AGCrA25, CGCrA25, GGCrA25, and UGCrA25, Supplemental Table 1), Quick CIP (NEB) were used to treat these oligos at a 100-μL reaction volume. Briefly, 5 μM of each 5′-triphosphate RNA oligo was incubated with 1× rCutSmart Buffer and 10 μL Quick CIP for 30 min at 37°C. The resulting product was purified by ethanol precipitation and dissolved in RNA storage buffer containing 10 mM MOPS pH 6 and 1 mM EDTA. The oligonucleotide concentration was estimated with a NanoDrop spectrophotometer (Thermo Fisher Scientific) and adjusted to 1 μM.

### Adding m^7^G cap to AGCrA25, CGCrA25, GGCrA25, and UGCrA25 RNA oligos

Capping of the 5′-triphosphate 28mer RNA oligos (AGCrA25, CGCrA25, GGCrA25, and UGCrA25, Supplemental Table 1) was carried out in 100-μL reactions using the Vaccinia Capping System (NEB). Briefly, 7 μM 5′-triphosphate RNA was mixed with 1× Capping Buffer (50 mM Tris-HCl, 5 mM KCl, 1 mM MgCl_2_, and 1 mm DTT, pH 8, at 25°C), 0.5 mM GTP, 0.1 mM SAM, and 50 units of Vaccinia Capping Enzyme and incubated overnight at 37°C. Capped RNAs were purified by electrophoresis in a 12% denaturing urea (7M) polyacrylamide gel. The purified capped RNAs were dissolved in RNA storage buffer (10 mM MOPS pH 6 and 1 mM EDTA), and their concentrations were estimated with the NanoDrop spectrophotometer and adjusted to 1 μM.

### RNA-seq library preparation for Illumina sequencing

*Experiments using SIRV RNA:* All sequencing experiments used a two-step procedure that separates the NTA addition and primer extension reactions by MRT and uMRT. For this procedure, 1 µL of the diluted SIRV-Set 3 (Lexogen) RNA was mixed with 0.5 μL of 5 μM MRT_dT18_opt primer (Supplemental Table 1) and 0.25 µL equimolar dNTP mix (10 mM each) by heating up to 95°C for 30 sec and then cooling down on ice. The reverse transcription was assembled by mixing the annealed primer/templates with 250 nM MRT or uMRT and the optimal reverse transcription buffer (50 mM Tris-HCl pH 8.3, 200 mM KCl, 2 mM MgCl_2_, 5 mM DTT, and 20% glycerol) to prepare a 5-µL reaction. After incubating the reaction at 42°C for 60 min, the reaction volume was adjusted to 10 µL with the composition of 750 nM MRT or uMRT, 1.25 mM dATP and 0.25 mM for the other three dNTPs, 1 μM TSO (MRT_TSO_5ab, Supplemental Table 1), 50 mM Tris-HCl pH 8.3, 100 mM KCl, 2 mM MgCl_2_, 5 mM DTT, 20% glycerol, and 12% PEG4000. Additional 30 min incubation at 42°C was followed. Then MRT or uMRT was inactivated by incubation at 80°C for 5 min. To prepare the cDNA libraries using universal human reference RNA (Thermo Fisher, Cat# QS0639), the RNA was diluted to 2 ng/µL. One microliter of the diluted RNA was used as the templates for cDNA library preparation by uMRT as done with SIRV-Set 3 RNA.

*Experiments using total human reference RNA*: To prepare the cDNA library for SSII (Thermo Fisher), 1 µL of 2 ng/µL universal human reference RNA (Thermo Fisher QS0639) was mixed with 0.5 μL of 5 μM MLV_dT30 primer (custom synthesis by Thermo Fisher) (Supplemental Table 1) and 0.5 µL equimolar dNTP mix (10 mM each) by heating up to 95°C for 30 sec and then cooling down on ice. The reverse transcription and template-switching reactions were assembled by mixing the annealed primer/templates with 0.5 µL SSII, 1 μM MLV_TSO (custom synthesis by IDT) (Supplemental Table 1), 2 µL 5× reaction buffer and nuclease-free water to prepare a 10 µL reaction. The reaction was incubated at 42°C for 90 min, and SSII was inactivated by incubating at 95°C for 2 min.

In the next step, the cDNA was preamplified by PCR using KAPA HiFi HotStart DNA polymerase (Roche). Three microliters of the cDNA product was used to prepare a 30-μL PCR reaction following the protocol by the manufacturer using a single PCR primer AmpPCR for uMRT cDNA libraries and mlvPCR (custom synthesis by Thermo Fisher) for SSII cDNA libraries (Supplemental Table 1) at a final concentration of 2 μM. The reaction was incubated at 98°C for 2 min, then cycled 16 times between (98°C 15 sec, 62°C 30 sec, 72°C 6 min), with a final extension at 72°C for 5 min. The PCR product was purified using AMPure XP beads (Beckman Coulter) by adding a 0.8× bead to sample ratio following manufacturer's protocol, and the concentration was determined by a Qubit High-Sensitivity DNA kit (Thermo Fisher).

To prepare the sequencing library, 0.25 ng of amplified cDNA was used for the tagmentation reaction carried out with a Nextera DNA Sample Preparation kit (Illumina), with the addition of 2 μL of a 2× Tagment DNA Buffer and 1 μL of Tagment DNA Enzyme, in a final volume of 4 μL. The tagmentation reaction was incubated at 55°C for 10 min and stopped by adding 1 μL Neutralize Tagment (NT) Buffer (Illumina). The whole volume was then used for limited-cycle enrichment PCR, along with 3 μL of Nextera PCR Mix (NPM), 1 μL of Index 1 primers (N7xx), and 1 μL of Index 2 primers (S5xx). The PCR reaction was incubated at 72°C 3 min, 95°C 30 sec, then 13 cycles between (95°C 10 sec, 55°C 30 sec, 72°C 30 sec), with a final extension at 72°C for 5 min. The PCR products were purified using AMPure XP beads by adding 1× bead to sample ratio following manufacturer's protocol. The libraries were checked for quality on a High-Sensitivity DNA chip by BioAnalyzer 2100 (Agilent), and quantification was done with a Qubit High-Sensitivity DNA kit (Thermo Fisher). Multiplexed sequencing libraries were pooled and sequenced using a NextSeq 500/550 platform (Illumina) using a 150 mid-output kit.

### RNA-seq library preparation for Oxford nanopore sequencing

To prepare the mRNA for nanopore sequencing library construction, Huh7.5 cells were grown in monolayer in a culture dish, and total cellular RNA was then extracted using TRIzol reagent (Thermo Fisher) by following the manufacturer's instruction. The mRNA from Huh7.5 cell total cellular RNA was isolated using NEBNext Poly(A) mRNA Magnetic Isolation Module ( E7490S) according to manufacturer's instructions. Then 10 ng Huh7.5 mRNA was used to prepare the cDNA libraries using the uMRT Template Switching Kit from RNAConnect (R1004S) according to the manufacturer's protocol.

To prepare the cDNA library for Maxima H minus (Thermo Fisher), 2 µL of 5 ng/µL Huh7.5 mRNA was mixed with 1 μL of 5 μM MLV_dT30 primer (Supplemental Table 1) and 1 µL equimolar dNTP mix (10 mM each) by heating up to 95°C for 30 sec and then cooling down on ice. The combined reverse transcription and template-switching reaction were set up by mixing the annealed primer/templates with 1 µL Maxima H minus, 1 μM MLV_TSO (Supplemental Table 1), 4 µL 5× reaction buffer, and nuclease-free water to prepare a 20-µL reaction. The reaction was incubated at 50°C for 90 min, and Maxima H minus was inactivated by incubating at 95°C for 2 min.

The resulting cDNA libraries were preamplified by PCR using KAPA HiFi HotStart DNA polymerase (Roche). A total of 20 μL of the unpurified cDNA product was used to prepare a 100-μL PCR reaction following the protocol by the manufacturer using a single PCR primer AmpPCR for uMRT cDNA libraries and mlvPCR for Maxima cDNA libraries (Supplemental Table 1) at a final concentration of 2 μM. The reaction was incubated at 98°C for 2 min, then cycled 12 times between (98°C 15 sec, 62°C 30 sec, 72°C 6 min), with a final extension at 72°C for 5 min. The PCR product was purified using AMPure XP beads by adding 1× bead to sample ratio following manufacturer's protocol. The concentration was determined by a Qubit High-Sensitivity DNA kit (Thermo Fisher), and the cDNA length distribution was analyzed by Agilent BioAnalyzer 2100.

To prepare the nanopore sequencing libraries, ∼50 fmol of the preamplified cDNA libraries were prepared for Nanopore sequencing using a Native Barcoding and Oxford Nanopore Ligation Sequencing Kit according to manufacturer's protocol. A unique barcode was used for each sample. Final libraries were pooled and sequenced on a FLO-MIN114, R10 flow cell in a MinION device, using MinKNOW software 24.02.8 for device control and data collection. Output was base-called using Dorado (7.3.11) with the Super High Accuracy model.

### Data analysis for ERCC quantification

To quantify the ERCC transcripts with sequencing data, the fastq files were aligned to the templates using Bowtie2 (version 2.4.2) with options *–local*, *‐‐no-unal*, and *–no-mixed* activated. The resulting SAM files were used to count the reads aligned to each ERCC transcript with a custom python script (see Supplementary Python scripts), and then the read count was normalized to obtain the TPM value (Zhao et al. 2021).

### Short-read sequencing data analysis

Paired-end sequencing reads were first trimmed with Trimmomatic (v0.40) to remove adapter sequences, and the trimmed reads were subsequently mapped to the human reference GRCh38 (RefSeq GRCh38.p14, GCF_000001405.40, primary assembly) with STAR (v2.7.11b). Mapped reads were quantified for each gene using featureCounts (v2.0.6) in paired-end mode without allowing overlap. The resulting raw counts per gene were transformed into TPM using a custom R script. Pairwise Pearson correlations between replicates and samples (uMRT or SSII) were calculated based on log2(TPM + 1). Genes were deemed as detected with at least one count in a sample. The distribution of reads across different genomic features (CDS, 5′UTR, 3′UTR, introns, TSS_up_10kb, TES_down_10kb) was calculated using the RSeQC software package (v5.0.1). Interestingly, we observed an increase in unannotated reads in uMRT when using the GENCODE 45 human reference. To investigate the effect of different sequencing depths an in silico read downsampling experiment was conducted. Paired reads for every sample were downsampled to 20 million using Seqtk (v1.4), and analyzed as described to assess read number of detected genes.

### Nanopore sequencing data analysis

Sequencing reads were initially trimmed to remove sequencing adapters and TSO sequences using Porechop (v0.2.4) with the ‐‐discard-middle option. The trimmed reads were then mapped to the human reference genome GRCh38 (RefSeq GRCh38.p14, GCF_000001405.40, primary assembly) using minimap2 (v2.28). Subsequently, gene quantification was performed on the mapped reads using IsoQuant (v3.4.1) with the preset ‐‐data_type nanopore. Pairwise Pearson correlations of gene expression between replicates and samples (uMRT or Maxima) were calculated based on log2(TPM + 1) values derived from the TPM output of IsoQuant. The R package Bambu (v3.0.8) was used to quantify isoform level expression with the discovery parameter set to “FALSE,” yielding counts per million (CPM) values for annotated isoforms. Isoforms were considered detected if they had at least one count in a sample. Similar to the analysis conducted with short-read data, the impact of varying sequencing depths on gene detection was evaluated through in silico read downsampling to 1.2 million reads, following the methodology described in the short-read methods. Pairwise Pearson correlations for isoform expression between samples (uMRT or Maxima) and replicates were calculated based on log2(CPM + 1) values. Sequencing read length was determined by samtools (v1.3.1).

### Data and data analysis script availability

The sequencing data are available under BioProject PRJNA1090626. Short-read and long-read data script that support the analysis in this manuscript is available on Zenodo: 10.5281/zenodo.12783273.

## SUPPLEMENTAL MATERIAL

Supplemental material is available for this article.

## COMPETING INTEREST STATEMENT

L.T.G., A.M.P., B.G., and A.E.S. are cofounders of RNAConnect, a company that is developing MRT as a reagent for sequencing and other applications. L.T.G. and A.M.P. are inventors on patents filed by Yale University for applications and adaptations of the MRT enzyme.

## Figures and Tables

**Li-Tao Guo:**
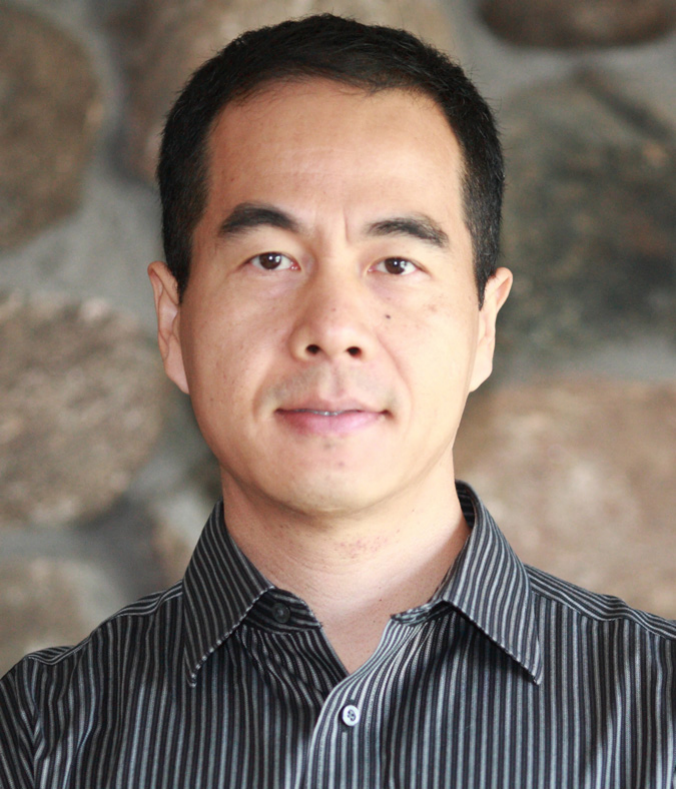


## MEET THE FIRST AUTHOR

***Meet the First Author(s)* is an editorial feature within *RNA*, in which the first author(s) of research-based papers in each issue have the opportunity to introduce themselves and their work to readers of *RNA* and the RNA research community. Li-Tao Guo is the first author of this paper, “Characterization and implementation of the MarathonRT template-switching reaction to expand the capabilities of RNA-seq.” Li-Tao did this work as an associate research scientist in Anna Pyle's lab at Yale University. He is now the Vice President of RNAConnect Inc., a new biotech company, where he leads research and development efforts focused on providing advanced tools for RNA research.**

**What are the major results described in your paper and how do they impact this branch of the field?**

In 2018, an ultraprocessive group II intron-encoded reverse transcriptase named MarathonRT was discovered in Anna Pyle's lab at Yale University. This enzyme exhibits powerful RNA structure unwinding activity and provides end-to-end coverage of highly structured, long RNA for cDNA synthesis, making it an ideal tool for RNA research. In this study, we carefully characterized each step of MarathonRT's template-switching activity and optimized the reaction conditions. By implementing a fully optimized procedure, we successfully harnessed the template-switching activity of MarathonRT, along with its enhanced variant UltraMarathonRT, to construct RNA-seq libraries. The use of MarathonRT and UltraMarathonRT in RNA-seq enables enhanced detection of structured or long RNA, thereby facilitating biomedical studies and biomarker discovery.

**What led you to study RNA or this aspect of RNA science?**

I have long been passionate about developing biotechnology to advance cutting-edge RNA research. RNA is the central biomolecule in human health and disease, but it is notoriously difficult to study due to its varying lengths, abundance, and tendency to fold into stable secondary and tertiary structures, which present significant challenges to commercially available reverse transcriptases. The discovery of MarathonRT marks a breakthrough that will revolutionize our ability to monitor the abundance and identity of individual RNAs within complex mixtures. I was fortunate to join the Pyle lab, where I focused on developing MarathonRT into a tool for RNA research.

**During the course of these experiments, were there any surprising results or particular difficulties that altered your thinking and subsequent focus?**

Template-switching capability is very rare among reverse transcriptases, so we were both surprised and excited to discover that MarathonRT possesses this activity. Previously, only MMLV RT and its variants were known to exhibit this capability.

**Are there specific individuals or groups who have influenced your philosophy or approach to science?**

Throughout my scientific career, I have been fortunate to work under the guidance of Youxin Jin, Dieter Söll, and Anna Pyle. Each of them has imparted invaluable lessons on the importance of independence, rigor, and critical thinking. My PhD advisor, Youxin Jin, emphasized the need to be quantitative whenever possible, laying the foundation for my critical thinking skills. Dr. Söll, my postdoc advisor, encouraged me to think outside the box, leading to many surprising discoveries during my research. Dr. Pyle taught me to remain critical, even of well-established results, which played a crucial role in advancing my research with MarathonRT.

**What are your subsequent near- or long-term career plans?**

The discovery of MarathonRT and the development of its applications led to the founding of RNAConnect, a biotech company where I now lead research and development. Our long-term goal is to provide a broad range of tools centered around MarathonRT, which can be used by the biomedical and biotechnology communities to advance RNA research.
